# Geographic Variation and Quality Consistency of Toddaliae Asiaticae Radix: A Hybrid Framework Integrating Environmental Feature and Bioactivity-Weighted Modeling

**DOI:** 10.3390/metabo16060353

**Published:** 2026-05-25

**Authors:** Linjiang Wei, Hong Chen, Mengmeng Sun, Yuanle Song, Chen Zhang, Zhi Zhou

**Affiliations:** Key Laboratory of Mass Spectrometry Imaging and Metabolomics, College of Life and Environmental Sciences, Minzu University of China, Beijing 100081, China; 24302658@muc.edu.cn (L.W.); zylch0221@163.com (H.C.); 24302648@muc.edu.cn (M.S.); 22302484@muc.edu.cn (Y.S.); 201801012@muc.edu.cn (C.Z.)

**Keywords:** Toddaliae Asiaticae Radix (TA), targeted analytical method, geographical factors, network pharmacology, quality consistency, machine learning

## Abstract

**Background**: Toddaliae Asiaticae Radix (TA) boasts a long history of medicinal application. However, origin traceability and quality assessment of the widely distributed original plant *Toddalia asiatica* have not been fully elucidated. **Methods**: A hybrid framework integrating targeted metabolomics, network pharmacology (NP), and machine learning (ML) was established. By optimizing key parameters, a high-coverage and rapid method for multiple categories compounds was developed using ultra-high performance liquid chromatography-multiple reaction monitoring tandem mass spectrometry (UPLC-MRM MS/MS). Using samples collected across 16 geographical regions, redundancy analysis (RDA) and pattern recognition techniques were applied to explore environment-sensitive metabolites. Taking into account five types of diseases, NP analysis was employed to obtain the bioactive components and their contribution weight in disease treatment. Subsequently, core Quality Markers (Q-Markers) with dual functions of responsive to geographic variations and biologically relevant to therapeutic efficacy were figured out, and were used to establish origin scoring model and discrimination model. **Results**: The geographical metabolic characteristics of the TA from broad regions in China were thoroughly analyzed, and 60 geographically sensitive compounds were identified. Through NP analysis, 27 core Q-Markers were locked. The bioactivity-weighted scoring model based on Q-Markers revealed the consistency of regional rankings as well as minor fluctuations across five diseases. ML demonstrated that the Q-Markers preserved regional discrimination performance, and the introducing of practical-oriented weights enhanced overall discriminative confidence. **Conclusions**: This research decodes the Geographical metabolic characteristics of TA, and highlights the necessity of function-oriented prioritization of drug resources.

## 1. Introduction

The medicinal plant *Toddalia asiatica* (L.) Lam. is the sole species in the genus Toddalia within the Rutaceae family [1]. The dried root or root bark of this plant, commonly used as a traditional folk medicine known as Toddaliae Asiaticae Radix (hereinafter referred to as TA), has been widely used among the ethnic minorities in southwestern China and is included in local medicinal standards [2]. TA exhibits multiple pharmacological properties, including analgesic effects, promotion of blood circulation to resolve stasis, and dispelling wind to alleviate discomfort [3,4]. Consequently, it is frequently used in the treatment of rheumatoid arthritis (RA) and venomous snake bites [5,6]. Modern pharmacological and pharmacodynamic studies have further demonstrated its significant spasmolytic, antimalarial, antibacterial, and antihyperlipidemic activities [7]. However, the medicinal value of TA remains underrecognized in rural regions, where the plant is often harvested for non-medicinal purposes such as crafting walking sticks and pipes. With the ongoing modernization of traditional Chinese medicine (TCM), interest in TA has grown due to its notable bioactivities and promising economic potential. Consequently, quality control and rational utilization of its plant resources have become critical research priorities.

TA contains a diverse array of bioactive constituents with varied chemical structures, and more than 165 individual compounds have been identified to date. Among these, alkaloids and coumarins are the most abundant [8,9], along with flavonoids, triterpenes, and phenolic acids [10,11,12]. Toddaculin has been proposed as a potentially active compound for the treatment of osteoporosis (OS) in TA [13]. Chelerythrine, a major alkaloid with diverse biological activities, also inhibits proliferation and induces apoptosis in human hepatocellular carcinoma (HCC) cells [14]. However, most studies on chemical composition have relied on ethanol, methanol, or chloroform extracts [15,16,17], whereas water-based extracts remain relatively understudied. As a classic and widely used preparation in traditional medicine, decoction more closely reflects actual clinical application and daily medication practices for this medicinal plant. Consequently, investigating aqueous extracts can better represent its true medicinal properties and clinical efficacy, underscoring the necessity and significance of the present study.

The quality and therapeutic efficacy of TCM is closely linked to their chemical profiles, which are modulated by origin-specific habitats that regulate bioactive constituent biosynthesis and accumulation [18,19]. Notably, the plant resources of TA are widely distributed across southern and southwestern China in diverse habitats ranging from low-altitude hills to high-elevation mountains and from humid river valleys to arid slopes [20], showing marked geographical variation in quality; yet current market evaluation standards remain inadequate [21]. Current quality standards for TA specify only chelerythrine as a marker compound. Several studies have employed high-performance liquid chromatography-ultraviolet (HPLC-UV), ultra performance liquid chromatography-tandem mass spectrometry (UPLC-MS/MS), and chemometric approaches to develop quantitative methods for additional constituents [22]. And the targeted analysis method based on multiple reaction monitoring (MRM) effectively expands the coverage of the target substances and reduces the interference from co-eluted compounds [23].

In recent years, machine learning (ML) models have been increasingly applied to classify and predict the geographical origins of TCM materials, with research advancing toward deep learning and multimodal data fusion. Notable applications include the integration of two-dimensional liquid chromatography coupled with Q-Orbitrap mass spectrometry (2D-LC-Q-Orbitrap-MS) with ensemble prediction for Huanglian classification [24], and the use of untargeted metabolomics combined with support vector machine models to identify *Acorus tatarinowii* with 100% accuracy [25]. However, current geographical origin evaluation mainly relies on chemical fingerprinting, yet most approaches neglect functional links between differential metabolites and therapeutic potency. Network pharmacology (NP), a pivotal tool in TCM modernization research, facilitates the mapping of relationships between bioactive components and diseases, and aids in identifying core component groups by predicting therapeutic targets [26]. NP-based studies elucidated its anti-thrombotic mechanism by inhibiting coagulation factor III via the PI3K-Akt-NF-κB signaling pathway [27]. TA also has potential for treating ischemic stroke (IS) through multiple components, targets, and pathways [28].

In this study, we propose a hybrid framework integrating environmental feature and bioactivity-weighted modeling to analyze origin-dependent differences in TA. Through extensive sampling across 10 provinces, a broad-coverage targeted analytical method was developed for multiple known components by meticulously optimizing LC elution conditions and MRM scan parameters. After acquiring metabolic profiles, multivariate statistical analyses were applied to assess the influence of geographical factors such as longitude, latitude, and altitude, and to screen out geo-sensitive chemical components. Subsequently, chemical components with potential disease-treating effects were elucidated using NP methods, and a unique Quality Marker (Q-Marker) subset that is both chemically responsive to geographic variations and biologically relevant to therapeutic efficacy was isolated using Venn analysis. A multi-index linear weighted comprehensive scoring model based on Q-Markers was then established. This model takes into account both the abundance of the compounds and their topological importance, providing preliminary insights into resource screening from different disease perspectives. Finally, ML approaches were used to verify the rationality of the 27 Q-markers obtained through biologically guided dimensionality reduction. This work provides a strategy and methodological reference for function-oriented geographical quality evaluation of TA.

## 2. Materials and Methods

### 2.1. Samples and Reagents

**Figure 1:**
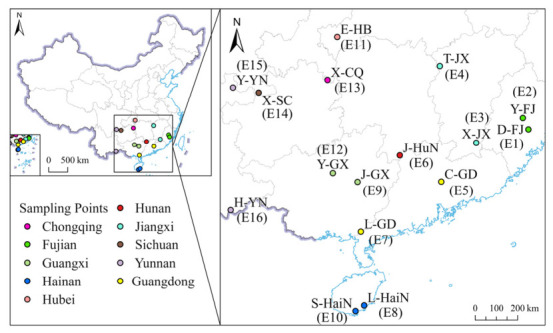
Map of plant *Toddalia asiatica* (L.) Lam. collection locations.This map is derived from the standard map service system of the Ministry of Natural Resources of China (http://bzdt.ch.mnr.gov.cn/index.html, accessed on 15 December 2024) and has been approved with the review number GS(2023)2767.

Acetonitrile, methanol, and formic acid (LC grade) were purchased from Thermo Fisher Scientific (Waltham, MA, USA). The deionized water used in the experiments was Wahaha pure water, obtained from Hangzhou Wahaha Group Co., Ltd. (Hangzhou, China). All specimens of the medicinal plant were collected and authenticated morphologically by professional technicians from Bozhou Zhiyao Agricultural Technology Co. Ltd. (Bozhou, China), a professional enterprise engaged in medicinal plant identification. This collection was conducted from April to July 2024 with matching growth status, covering 16 locations in 10 provincial administrative regions of China (n = 3, denotes three independent wild individual plants). The sampling sites were shown in Figure 1, and detailed information can be found in Supporting Information Table S1. Map data was sourced from the Standard Map Service System of the Ministry of Natural Resources of China (review number: GS(2023)2767, http://bzdt.ch.mnr.gov.cn/index.html, accessed on 15 December 2024).

### 2.2. Sample Preparation

Fresh roots were transported to the laboratory in sealed bags and stored at −20 °C prior to pretreatment. The roots were thoroughly rinsed with water, cut into small pieces, and air-dried in a cool, well-ventilated room. The dried materials were ground into a fine powder and passed through a 40-mesh sieve, then stored in sealed containers at 4 °C in the dark until further analysis. Exactly 10.0000 g was weighed and placed in a round-bottom flask for reflux extraction using 100 mL of water for 1 h, repeated twice. The resulting two-part extract was filtered and combined, then freeze-dried into powder after rotary evaporation concentration for storage. For instrument analysis, 0.0200 g of dried powder was precisely weighed and redissolved in 400 μL of water. After centrifugation at 12,000 rpm for 10 min, the supernatant was filtered through a 0.22 μm membrane.

Quantification of 80 analytes was performed using a relative quantitative metabolomics method, which was performed according to our previously reported method [29,30]. A reference sample was prepared by mixing equal aliquots of TA powder from multiple origins. The concentration level of the sample obtained by resolubilizing 0.2 g of the mixed powder in 400 μL of water was defined as 1C (where “C” represents the original concentration in reference sample). Accordingly, a reference sample at a concentration level of 4C was prepared by resolubilizing the same amount of powder in 100 μL of water. The 4C sample was then serially diluted to obtain samples with relative concentrations of 2C, 1C, 0.5C, 0.1C, 0.005C, 0.001C, 0.0005C, and 0.0001C for the development and validation of the analytical method.

### 2.3. UPLC-MRM MS/MS Analysis

An Exion LCTM chromatography system (Shimadzu, Japan) coupled with a quadrupole-linear ion trap mass spectrometer (QTRAPTM 6500, SCIEX, Framingham, MA, USA) was used for LC-MS/MS analysis. LC separation was performed using a Waters ACQUITY UPLC T3 column (2.1 mm × 100 mm, 1.8 μm, Waters Corp., Milford, MA, USA), with the column temperature maintained at 30 °C. The mobile phases consisted of 0.1% formic acid aqueous solution (A) and acetonitrile (B). The injection volume was 5 μL. Based on criteria of rapid and efficient separation, elution flow rates of 0.2 mL/min, 0.25 mL/min, and 0.3 mL/min were evaluated and optimized, with corresponding adjustments to the elution gradient and time. The final optimized gradient elution conditions were as follows: 0–2 min, 2–8% B; 2–4 min, 8–25% B; 4–9 min, 25–30% B; and 9–17 min, 30–70% B, with a flow rate of 0.25 mL/min. Complete details of the gradient conditions investigated are provided in Section S1.

The mass spectrometer was equipped with an ESI probe and operated in positive ion mode using MRM scan mode. The operating parameters were as follows: electrospray voltage, 5500 V; nebulizer gas (GS1), 55 psi; auxiliary gas (GS2), 50 psi; curtain gas (CUR), 20 psi; and source temperature, 450 °C. MRM ion pairs for the target metabolites were selected based on prior high-resolution MS analysis. Declustering potential (DP) and collision energy (CE) for each ion pair were optimized to establish the targeted quantitative analysis method. DP values were evaluated at 50 V, 70 V, 90 V, and 110 V, while CE values were investigated at 20 eV, 25 eV, 30 eV, 35 eV, 40 eV, 45 eV, 50 eV, 55 eV, and 60 eV.

### 2.4. Method Performance Testing

The linearity, precision, accuracy, and sample stability of the newly established method were investigated. For the linearity assessment, samples at each concentration level were injected and analyzed five times, and calibration curve fitting was performed to obtain the equation and correlation coefficient (r). For precision evaluation, a sample at the 1C concentration level was injected six times daily over three consecutive days. The relative standard deviation (RSD) values of the target component contents were calculated to assess intra-day and inter-day precision. To evaluate accuracy, three concentration levels of the test solution were used, as follows: low (0.5C), medium (1C), and high (2C). Each level was injected in parallel six times, and the responses were substituted into the calibration curve to calculate the relative error (RE) values. Sample stability over a 72 h period at 4 °C was additionally evaluated by calculating the response deviation.

### 2.5. Data Processing and Mining

Peak integration and relative quantification were performed using MultiQuant software (version 3.0.2, Sciex, Framingham, MA, USA). Orthogonal partial least squares discriminant analysis (OPLS-DA) was conducted using SIMCA-P (version 14.1, Umetrics, Umeå, Sweden) to explore differences in chemical composition among samples from different origins. The OPLS-DA models were verified through cross-validation and 200 permutations. The selection of differential variables followed the conventional standards in metabolomics, such as VIP > 1, within-group RSD controlled at 50%, and FDR corrected t-tests etc. Heatmaps were generated using the R language (version 4.3.2). To investigate the influence of latitude, longitude, and altitude on each component, redundancy analysis (RDA) was performed using an online tool (http://cloudtutu.com.cn/).

In addition, a “components–targets—diseases” network was constructed using NP analysis. SMILES information of known components was obtained from PubChem (https://pubchem.ncbi.nlm.nih.gov/), and uploaded to the Swiss Target Prediction database (http://swisstargetprediction.ch/) to identify potential target proteins. The GeneCards database (https://www.genecards.org/) was then used to retrieve disease-related targets associated with TA, including RA, OS, ischemic stroke (IS), hyperlipemia (HY), and HCC. Common targets between the constituents and specific diseases were identified using the Venny online platform (https://bioinfogp.cnb.csic.es/tools/venny/index.html, accessed on 22 December 2024), and STRING (https://www.string-db.org/) was subsequently used to construct a protein–protein interaction (PPI) network for core targets screening. Topological parameters—including degree, closeness, and betweenness—were calculated to reflect the importance of each key component. The network diagram was visualized using Cytoscape (version 3.9.1).

## 3. Results

### 3.1. Analytical Performance and Validation of the Relative Quantitative Method

Our research group previously conducted a chemical analysis of TA, obtaining MS/MS spectra for 64 major components and elucidating their fragmentation patterns. Building on this foundation, the present study selected these 64 known components along with 16 newly identified ones as target metabolites for a rapid quantitative method. Structurally, the target metabolites included 46 alkaloids, 9 coumarins, 6 organic acids, 5 flavonoids, and 14 other compounds (Table S2). Given that the previously reported LC method (published in Chinese) required 70 min per run, which is inefficient for routine analysis, UPLC-MRM MS/MS was employed in this study to optimize parameters for the rapid quantification of constituents across multiple categories.

To achieve better separation and MS response, the gradient elution conditions were systematically optimized. The following six gradients were evaluated: gradient 1 (28 min), gradient 2 (25 min), gradient 3 (15 min), gradient 4 (15 min), gradient 5 (17 min), and gradient 6 (17 min). Based on extracted ion chromatograms (EICs) (Figure S1), gradients 1 and 2 provided better resolution but resulted in longer analysis times and fewer peaks within the first 12 min, reducing overall efficiency. Gradient 3 had the shortest runtime; however, certain compound separations were inadequate due to peak overlap. At a total run time of 17 min, the separation of target compounds improved significantly. Considering the overall chromatographic separation and peak shape characteristics, gradient 6 was selected as the optimized condition.

**Figure 2:**
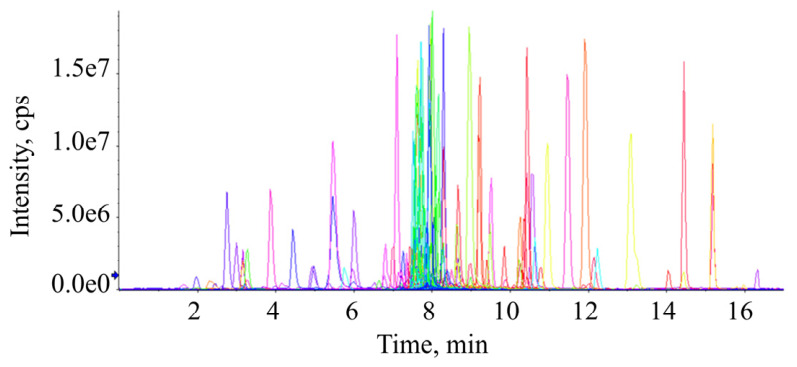
Representative extracted ion chromatogram of *Toddalia asiatica* aqueous extract using the optimized UHPLC-MRM MS/MS approach. Each colored trace corresponds to the MRM transition of a specific analyte (see Table S3 for details).

The flow rate was also examined at 0.2 mL/min, 0.25 mL/min, and 0.3 mL/min (Figure S2). A flow rate of 0.2 mL/min prolonged compound retention t and enhanced separation, but complete elution of all target compounds could not be achieved within 17 min. A flow rate of 0.3 mL/min reduced analysis time. In comparison, 0.25 mL/min offered superior balance between separation efficiency and coverage, making it more suitable for detecting most compounds. More importantly, the DP and CE values for each MRM ion pair were systematically optimized to ensure appropriate responses (Table S3). For low-abundance compounds, detection intensity should be maximized; however, this was not the sole criterion. For high-abundance compounds, responses were moderately attenuated to minimize dynamic range disparities among all targeted constituents. The representative EIC obtained using the optimized elution gradient, flow rate, and DP and CE values is shown in Figure 2.

**Figure 3:**
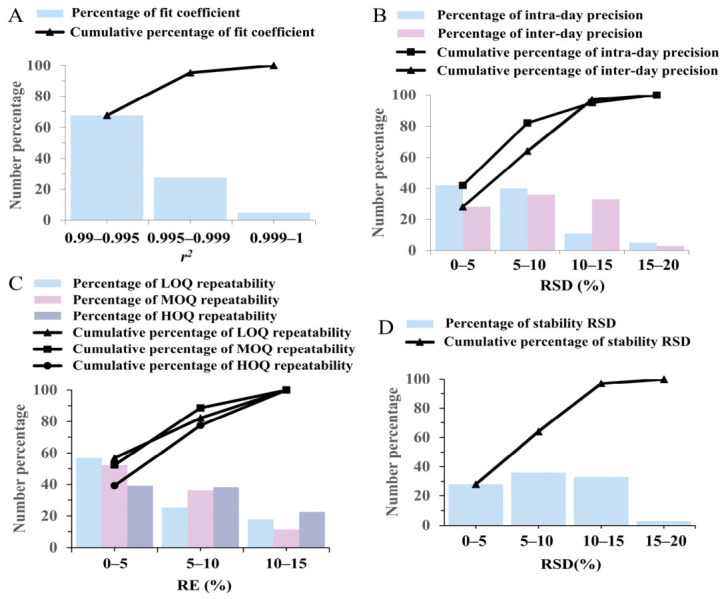
Method validation results for the rapid relative quantitative analysis of 80 multi-category compounds in Toddaliae Asiaticae Radix extract. (**A**) Distribution of r^2^ values for linearity; (**B**) Distribution of RSD for intra- and inter-day precision; (**C**) Distribution of RE for accuracy; (**D**) Distribution of RSD for sample stability.

The linearity, precision, accuracy, and stability of the newly established method were subsequently evaluated (Table S4). Linearity results indicated that all 80 components exhibited a strong linear relationship between relative concentration and instrument response within a defined range, with correlation coefficients (*r*^2^) exceeding 0.99 (Figure 3A). For precision, intra-day and inter-day RSD values for the 80 components were below 16.34% and 15.96%, respectively (Figure 3B). Accuracy assessments showed that the RE values at low, medium, and high concentration levels did not exceed 14.48%, 14.87%, and 14.83%, respectively (Figure 3C). Sample stability was assessed after storage at 4 °C for 72 h, during which variation in detection remained under 15.96% for all components (Figure 3D). In summary, the relative quantitative method demonstrated excellent linearity, precision within 20%, accuracy within 15%, and stable sample integrity throughout analysis—meeting requirements for bioanalytical method validation as recommended by the U.S. FDA (2018) guidance [31]. Furthermore, it enabled simultaneous quantification of multiple chemical components in TA extracts, offering broader coverage than existing methods that target typically only a few index compounds. The total analysis time was reduced to just 17 min, representing a significant improvement over the previous 70 min protocol.

### 3.2. Metabolomic Profiling and Geographical Variation in TA Across Different Origins

**Figure 4:**
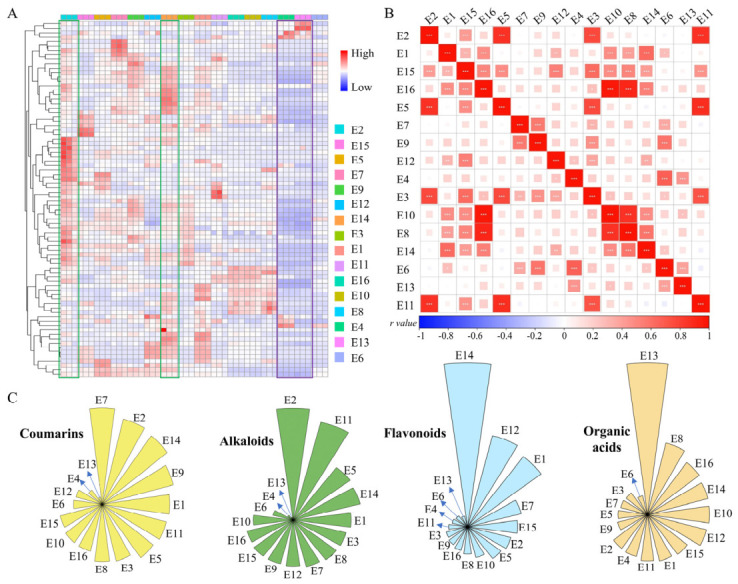
Variation in chemical components of Toddaliae Asiaticae Radix from 16 regions. (**A**) Cluster heatmap; (**B**) Pearson correlation diagram; (**C**) Separate ranking by component category. * *p* < 0.05, ** *p* < 0.01, *** *p* < 0.001.

The newly developed method was applied to analyze samples from 16 regions across 10 provinces, with two regions sampled in six of those provinces. The relative content of each analyte was calculated based on the peak area detected by LC-MRM MS/MS, combined with the standard curves established by gradient concentrations of the reference sample. First, a clustering heat map was generated based on compound quantitative data (Figure 4A). It was observed that the overall content of chemical components in E2 and E14 was notably high, whereas E6 and E13 showed substantially lower levels. Notably, E2 and E13 are geographically proximate but exhibit considerable altitudinal differences—1191 m and 631.5 m, respectively. These observations suggest that geographical factors may influence the chemical composition of TA.

This hypothesis was further supported by regional sample correlation analysis (Figure 4B). Except for the two Hainan Province sites, which showed a high correlation coefficient (*r* = 0.87), samples from the same province generally showed lower similarity. Unexpectedly, strong correlations were observed among E2, E3, E5, and E11, despite their non-adjacent geographical distributions. This observation suggests that specific shared environmental or geological characteristics, rather than simple spatial proximity, may dominate the metabolic similarity, which prompted further in-depth analysis of the geography-associated metabolic patterns.

When categorized by structural classes including coumarins, alkaloids, flavonoids, and organic acids (Figure 4C), the regional ranking of compound abundance varied. For instance, E7 had the highest coumarin levels, with E2 and E14 also ranking highly; E2 and E11 showed elevated alkaloid content; and E14 samples contained exceptionally high flavonoid concentrations. In contrast, levels of coumarin, alkaloids, and flavonoids were relatively low in E13, E4, and E6. However, E13 samples contained significantly higher levels of organic acids. These findings clearly indicate marked regional variation in the chemical composition of TA, suggesting a significant influence of geographical factors.

### 3.3. Identification of Q-Markers via Integrated Chemical Sensitivity and Biological Relevance

**Figure 5:**
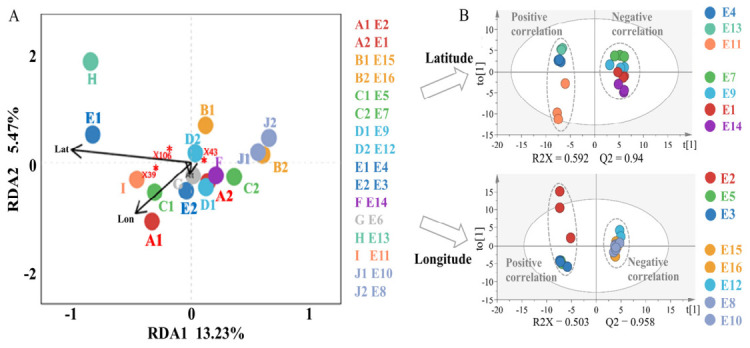
Influence of geographical factors on the chemical composition of Toddaliae Asiaticae Radix. (**A**) RDA incorporating latitude, longitude, and altitude; (**B**) OPLS-DA score plot identifying components correlated with latitude and longitude.

The effects of latitude, longitude, and altitude on samples from different regions were analyzed using RDA (Figure 5A). Among the three, latitude emerged as the most influential factor. The overall component concentrations in E4, E11, and E13 were positively correlated with latitude, with values of 28.58° N, 28.31° N, and 30.11° N. In contrast, samples from E1, E7, E9, and E14 were negatively correlated with latitude, corresponding to 21.81° N, 23.94° N, 25.5° N, and 27.84° N, respectively. For longitude, samples from E2, E3, E5, and E11 were aligned with the positive direction of the vector, spanning 109.37° E to 117.96° E. Conversely, samples from E8, E10, E12, E15, and E16 were oriented toward the negative direction, with longitudes ranging from 104.17° E to 110.03° E. Among the three factors, altitude showed the least influence on the chemical composition of the samples. Nevertheless, altitude had a positive impact on samples from E6 and a negative impact on those from E12.

Based on the observed relationships with geographical factors, OPLS-DA models were constructed to compare regional samples with positive and negative correlation (Figure 5B). All OPLS-DA models exhibited Q^2^ values above 0.5. Permutation validations yielded R^2^-intercept < 0.621 and Q^2^-intercept < −0.339, confirming the absence of overfitting (Figure S3). As a result, a total of 60 components was found to be sensitive to geographical factors, as follows: 26 correlated with latitude, 15 with longitude, and 39 with altitude (Table S5).

To further identify markers with both geographical discrimination capacity and biological relevance, NP analysis was performed on TA and its five related diseases, including RA, OS, IS, HY, and HCC, to explore the quantitative pattern of biological functional weights of metabolites, providing biological evidence for weight construction in the subsequent origin-evaluation model. In component–target–disease correlation networks, topological parameters reflect and help map the relative importance of each compound node for a given disease [32,33]. Degree, a topological parameter, represents the number of connections between a node and others; a higher value indicates a more critical position within the network. Betweenness reflects a node’s capacity to mediate information flow among other nodes. Closeness centrality measures the average distance from one node to all others, indicating functional efficiency in information disseminating or performing its function. Collectively, these parameters highlight the significance of each component in therapeutic effects, regulation of disease-related signaling pathways, and overall drug efficacy [34].

**Figure 6:**
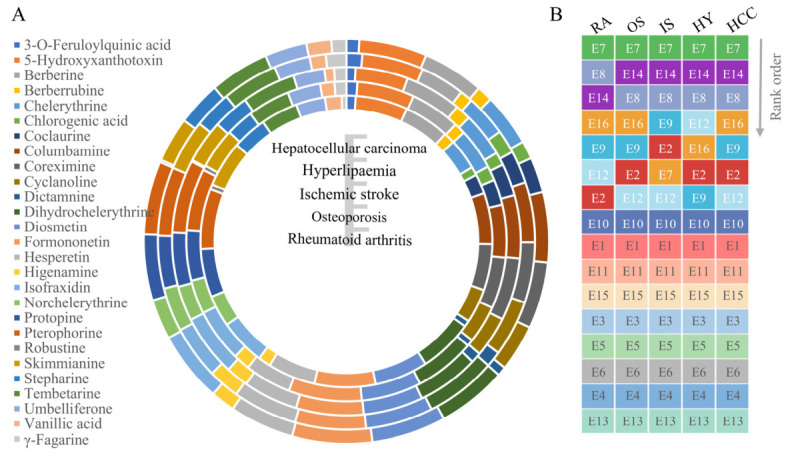
The multi-index scores model and corresponding rank orders for regions. (**A**) Comprehensive weights of topological parameters of components across different diseases; (**B**) Rank orders for 16 regions.

It should be noted that NP was used herein merely as a computational weighting tool for reducing subjective scoring biases, rather than for definitive mechanistic exploration. Accordingly, all 40 components with well-defined target information in public databases were included for weight calculation. Venn diagram analysis revealed 27 overlapping metabolites between the 60 geographically sensitive compounds and 40 target-annotated components, which were defined as core Q-Markers (Figure S4). Figure 6B illustrated that the topological importance of a single component varies depending on the disease. Furthermore, it was found that there are 11 compounds that meet the criteria of OB ≥ 30% and DL ≥ 0.18 among the 40 components by searching the TCMSP database. Among these, 9 were retained in the 27 Q-Markers (Table S6).

### 3.4. Construction of a Utility-Oriented Weighting Framework and Comparative Validation via ML

Given that conventional assessment methods consider only the content of a few components, we introduced topological parameters to establish a multi-index linear weighted comprehensive model. The core principle involved multiplying each component’s content by its topological weight within a specific disease network and summing the products to obtain a comprehensive score for each origin. The calculation formula is as follows:

(1) $${Score}_{j}=\sum \left({Weight}_{i}{\times Ingredient\;content}_{i,j}\right)$$

Here, subscript *j* denotes the place of origin, and *i* refers to the components. Weight represents the normalized sum of topological parameters, to eliminate dimensional bias. Normalization was performed as follows:

(2) $${NormPara}_{m,j}=({Para}_{m,j}\;-\;{MinPara}_{m,j})/{(MaxPara}_{m,j}\;-\;{MinPara}_{m,j})$$

Subscript *m* represents degree, betweenness, or closeness centrality. This model was implemented in Python 3.10.

The multi-index scores and corresponding rank orders for the 16 regions were subsequently computed (Figure 6). While the specific rankings exhibited minor fluctuations across the five disease models—reflecting the varying topological contributions of metabolites within different disease-specific networks—the overall distribution of top-tier and bottom-tier regions remained highly consistent. Notably, regions E7, E8, and E14 emerged as the highest-ranking origins across all evaluated contexts. Within the scope of this exploratory computational tool, these origins possess a higher integrated potential for providing bioactive-rich TA resources. These findings provide a preliminary geographical guide for the selection of high-quality medicinal resources, although further field-based studies are required to confirm the influence of specific environmental gradients on these quality profiles.

**Figure 7:**
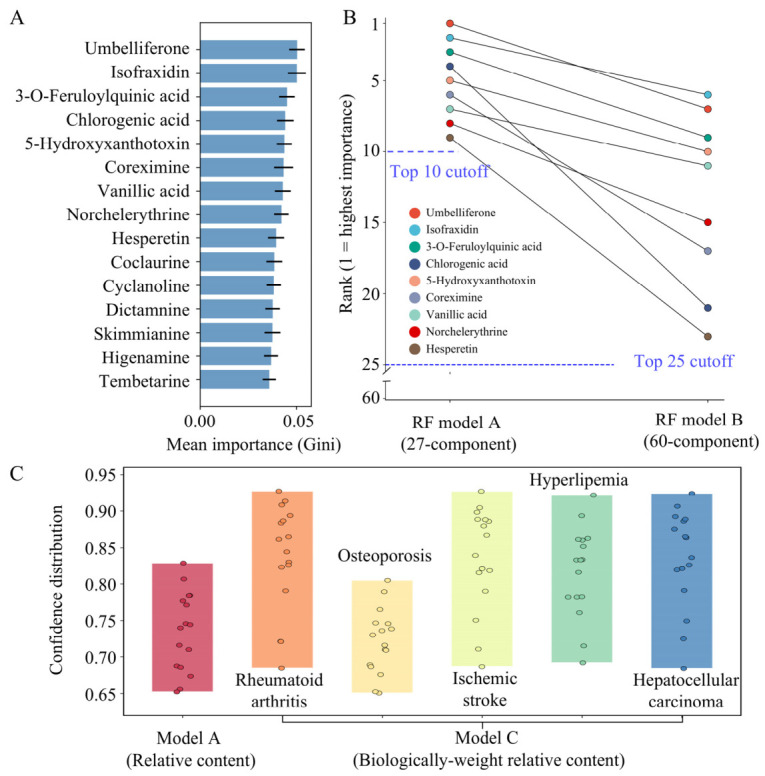
Comparative validation via machine learning model. (**A**) Feature importance analysis of bioactivity-weighted model based on 27 Q-Markers; (**B**) Consistency in feature importance ranking of overlapping metabolites between Model A and Model B; (**C**) Comparison of classification confidence intervals between Model A and Model C.

To further validate the rationality of screened core Q-Markers and the superiority of the bioactivity-weighted strategy, template-matching ML classification models were constructed for comparative verification. Through structured-data feature engineering, statistical features and pairwise proportion metrics were extracted from a 27-component dataset. PCA was applied for dimensionality reduction to preserve core data variations and optimize distance calculation for subsequent k-nearest neighbor (KNN) classification [35]. Weighted Euclidean, Manhattan, and cosine distance were calculated for KNN modeling, with leave-one-out cross-validation (LOOCV) used to assess model performance [36]. Random forest (RF) was used to explain the metabolite contribution in the origin classification model, and the 15 metabolites with the highest contribution rates were visualized (Figure 7A).

To rigorously evaluate the region discrimination ability of the Q-marks, a random mixing strategy was employed to construct a validation dataset. Specifically, individual samples from each region were randomly mixed to generate representative a simulated batch consisting of 16 samples. We first performed a feature-ablation analysis to compare the discriminative performance of the 27 refined Q-Markers (Model A) versus the 60 initial geographically sensitive compounds (Model B). Both models achieved 100% origin-classification accuracy and comparable F1-scores. The classification confidence intervals were 0.652–0.829 for Model A and 0.622–0.730 for Model B. Random-forest-derived feature-importance ranking revealed that top 9 compounds in Model A ranked in the top 25 in Model B (Figure 7B).

Furthermore, the classification performance of models built with relative content versus weighted-relative content (Model C) of 27 Q-Markers was compared. Model C included five disease weights and yielded five sets of performance data. The results showed that the classification of model C maintained a high performance with an accuracy of 93.75% and an F1 score of 0.917, involving only a single misclassified sample. The confidence ranges for the classification of the five diseases were as follows: 0.684–0.927 (RA), 0.651–0.805 (OS), 0.687–0.927 (IS), 0.692–0.922 (HY), and 0.684–0.924 (HCC). In summary, the inclusion of the biologically guided weight data has increased the confidence of regional classification (Figure 7C).

## 4. Discussion

The quantitative strategy adopted in this study is a broad-coverage relative quantitative method based on sample stepped dilution. It has previously been applied to the analysis of over 1000 metabolites in large-scale samples from plasma [29,30], which does not require the use of standard substances and can effectively improve data quality. Although absolute values of content cannot be obtained, relative abundance measurements also have high robustness for capturing systematic metabolic changes. In terms of geographical traceability, the ratios and fold changes of 80 targeted metabolites between samples provide a more comprehensive metabolic feature than the absolute concentrations of a few standard substances, which is pivotal for capturing the complex response of the TA metabolome to geographical shifts.

RDA revealed significant spatial differentiation of metabolites in TA, whose abundance was distinctly geographically correlated with latitude and longitude. It must be recognized that latitude and longitude can serve as comprehensive indicators of complex climate variables, such as temperature, ultraviolet (UV) radiation intensity, and precipitation patterns. Although our data show a strong statistical correlation, these findings should be understood as spatial distribution patterns rather than direct physiological evidence. Observations in low-latitude regions show an increase in the content of certain alkaloids, suggesting that the TA may have adaptive responses to the environment it inhabits. Alkaloids containing conjugated double bonds or aromatic rings usually play a defensive role with higher UV and more complex biological interactions by stimulating the phenylpropanoid pathway [37,38]. Similarly, the accumulation of chlorogenic acid and its analogues helps to reduce UV damage in low-latitude habitats, as well as herbivore damage and disease incidence [39,40,41,42].

The core of this study lies in exploring a quality assessment based on biological principles. The initial 60 geographically sensitive components were streamlined to 27 core Q-Markers through NP analysis, reducing data redundancy while enhancing functional relevance. KNN-based ML model proved that these Q-Markers had the same classification accuracy as the 60 components set. It demonstrated that biologically guided filtering reduced the dimensionality while fully preserving the geographical fingerprint of the medicinal resources. Additionally, the practical-oriented weighted model presented superior overall performance on classification confidence, which indicates incorporating biological weights may have helped optimize the mathematical structure of the data, thereby making regional clustering more evident. To ensure statistical robustness, 500-iteration permutation verification confirmed no overfitting despite the limited sample size (Figure S5).

In the scoring model, regional rankings remained broadly consistent across different diseases, with minor fluctuations only observed among top-ranked regions. Stable ranking of the lower-scoring regions indicates a benchmark effect, with relatively low abundance of Q-Markers in these sources regardless of the disease-specific weights. On the contrary, slight variations among higher-scoring regions may reflect the functional sensitivity of the weight framework. Due to the topological importance of metabolites changing according to pathological conditions, such fluctuations allow the model to discriminate chemical profiles suitable for specific therapeutic applications. Collectively, this comprehensive framework has the potential to become a precise and targeted priority selection tool, capable of identifying the most chemically suitable drug resources based on various clinical needs.

Finally, this framework should be viewed as an exploratory computational prioritization tool. While it effectively ranks origins based on current pharmacological knowledge, the predictive scores require further validation through in vivo bioassays. Future research integrating absolute quantification of key Q-Markers and multi-omics will provide a more granular understanding of the gene–environment interactions driving the quality of this valuable medicinal resource.

## 5. Conclusions

This study constructed a comprehensive scoring framework to bridge the gap between the geographical and chemical specificity differences of the TA and their therapeutic efficacy. Through the integration of broad-coverage targeted metabolomics and NP analysis, 27 core Q-Markers were identified, which are sensitive to geographical environmental factors and biologically related to clinical targets. Through ML validation, the robustness of the biologically guided feature selection was confirmed. The results showed that the optimized feature set retained a high discriminatory ability, and the improved overall classification confidence. Our results elucidate the regional differences of TA at the metabolic molecular level, and highlight the necessity of transitioning from the traditional content-based traceability to function-oriented prioritization of drug resources. Ultimately, this exploratory computational framework offers a flexible and scalable strategy for the quality assessment of multi-source medicinal plants, facilitating more precise utilization of traditional herbs.

## Data Availability

The data that support the findings of this study are available upon request from the corresponding author, upon reasonable request.

## Supplementary Materials

The following supporting information can be downloaded at: https://www.mdpi.com/article/10.3390/metabo16060353/s1, Section S1. Optimization of gradient elution conditions; Figure S1: Extracted ion chromatograms in optimization of gradient elution condition; Figure S2: Extracted ion chromatograms in optimization of flow rate; Figure S3: E The OPLS-DA models constructed for screening geographically sensitive chemical components and the corresponding results of permutation tests; Figure S4: The Venn diagram related to Q-Marker selection; Figure S5: Optimization and assessment of KNN based region classification model using 27 Q-Markers; Table S1: Detailed information of collection regions of medicinal plant *Toddalia asiatica* (L.) Lam; Table S2: Detail information of 80 target components in *Toddalia asiatica* for rapid quantitative analytical method; Table S3: The optimized parameters of UHPLC-MRM MS/MS method for targeted analysis of components in *T. asiatica*; Table S4: Methodological validation results of quantitative analysis for components in *T. asiatica*.

## Abbreviations

The following abbreviations are used in this manuscript:

CE	Collision energy
CUR	Curtain gas
DL	Drug likeness
DP	Declustering potential
EICs	Extracted ion chromatograms
GS1	Nebulizer gas
GS2	Auxiliary gas
HCC	Hepatocellular carcinoma
HPLC	High-performance liquid chromatography
HY	Hyperlipemia
IS	Ischemic stroke
KNN	K-nearest neighbor
LC-MS	Liquid chromatography-mass spectrometry coupling technique
LOOCV	Leave-one-out cross-validation
ML	Machine learning
MRM	Multiple reaction monitoring
MS/MS	Tandem mass spectrometry
NP	Network pharmacology
OB	Oral bioavailability
OPLS-DA	Orthogonal partial least squares discriminant analysis
OS	Osteoporosis
PCA	Principal component analysis
PPI	Protein–protein interaction
RA	Rheumatoid arthritis
RDA	Redundancy analysis
RE	Relative error
RF	Random forest
RSD	Relative standard deviation
TA	Toddaliae Asiaticae Radix
TCM	Traditional Chinese medicine
TCMSP	Traditional Chinese medicine systems pharmacology
UV	Ultraviolet
VIP	Variable importance in the projection
