# Supplementary material for: Geographic Variation and Quality Consistency of Toddaliae Asiaticae Radix: A Hybrid Framework Integrating Environmental Feature and Bioactivity-Weighted Modeling
Source: Metabolites. 2026 May 25;16(6):353. doi: 10.3390/metabo16060353 (PMC13304387; doi:10.3390/metabo16060353)
Supplement: Supplementary file 1 [file metabolites-16-00353-s001.zip › metabolites-4296735-supplementary.pdf]

Supplementary material

Geographic variation and quality consistency of Toddaliae Asiaticae Radix: a hybrid framework integrating environmental feature and bioactivity-weighted modeling

Linjiang Wei [a,b,&], Hong Chen [a,b,&], Mengmeng Sun [a,b], Yuanle Song [a,b], Chen Zhang [a,b,c], Zhi Zhou [a,b,c,]

[a] *College of Life and Environmental Sciences, Minzu University of China, Beijing 100081, P. R. China*

[b] *Key Laboratory of Mass Spectrometry Imaging and Metabolomics (Minzu University of China), National Ethnic Affairs Commission, Beijing 100081, P. R. China*

[c] *Key Laboratory of Ethnomedicine of Ministry of Education (Minzu University of China), Beijing 100081, China*

Table of Contents

Section S1. Optimization of gradient elution conditions

Figure S1. Extracted ion chromatograms in optimization of gradient elution condition

Figure S2. Extracted ion chromatograms in optimization of flow rate

Figure S3. The OPLS-DA models constructed for screening geographically sensitive chemical components and the corresponding results of permutation tests. (A) Latitude; (B) Longitude; (C) Altitude.

Figure S4. The Venn diagram related to Q-Marker selection. (A) Target intersect of 40 components in Toddaliae Asiaticae Radix (TA) and five diseases; (B) Intersect of 60 geographically sensitive components and 40 potential pharmacological active compounds.

Figure S5. Optimization and assessment of KNN based region classification model using 27 Q-Markers. (A) Optimization of K value; (B) Permutation verification with 500-iteration.

Table S1. Detailed information of collection regions of medicinal plant *Toddalia asiatica* (L.) Lam.

Table S2. Detail information of 80 target components in TA for rapid quantitative analytical method.

Table S3. The optimized parameters of UHPLC-MRM MS/MS method for targeted analysis of components in TA

Table S4. Methodological validation results of quantitative analysis for components in TA

Table S5. 60 chemical components in TA that are correlated with latitude, longitude and altitude.

Section S1. Optimization of gradient elution conditions

Gradient 1

A linear gradient elution was used as follows: 0–5 min, 2%–5% B; 5–8 min, 5%–10% B; 8–15 min, 10%–30% B; 15–20 min, 30%–40% B; 20–22 min, 40%–60% B; and 22–28 min, 60% B.

Gradient 2

A linear gradient elution was used as follows: 0–5 min, 2%–5% B; 5–8 min, 5%–10% B; 8–12 min, 10%–30% B; 12–17 min, 30%–40% B; 17–19 min, 40%–60% B; and 19–25 min, 60% B.

Gradient 3

A linear gradient elution was used as follows: 0–2 min, 1%–5% B; 2–4 min, 5%–20% B; 4–7 min, 20% B; and 7–15 min, 20%–65% B.

Gradient 4

A linear gradient elution was used as follows: 0–2 min, 1%–5% B; 2–4 min, 5%–20% B; 4–5 min, 20%–30% B; 5–9 min, 30%–40% B; and 9–15 min, 40%–65% B.

Gradient 5

A linear gradient elution was used as follows: 0–2 min, 1%–5% B; 2–4 min, 5%–20% B; 4–4 min, 20%–30% B; 7–15 min, 30%–40% B; and 15–17 min, 40%–65% B.

Gradient 6

A linear gradient elution was used as follows: 0–2 min, 2%–8% B; 2–4 min, 8%–25% B; 4–9 min, 25%–30% B; and 9–17 min, 30%–70% B.

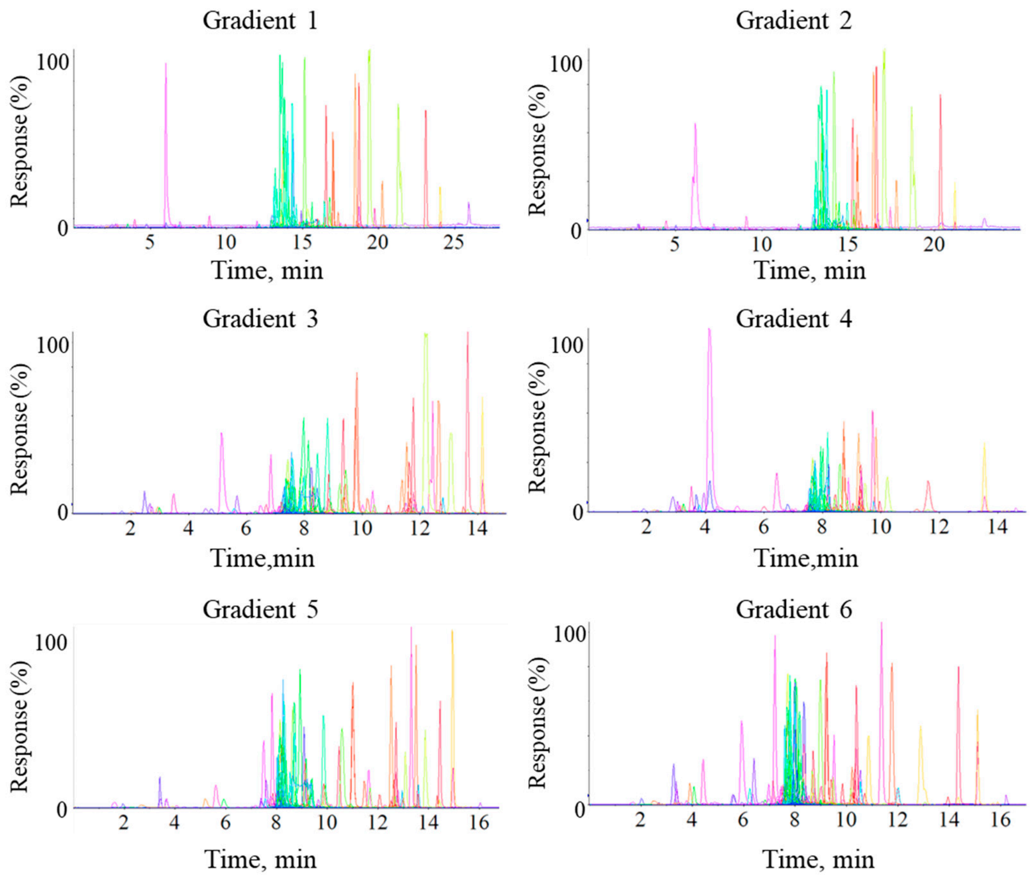

Figure S1. Extracted ion chromatograms in optimization of gradient elution condition

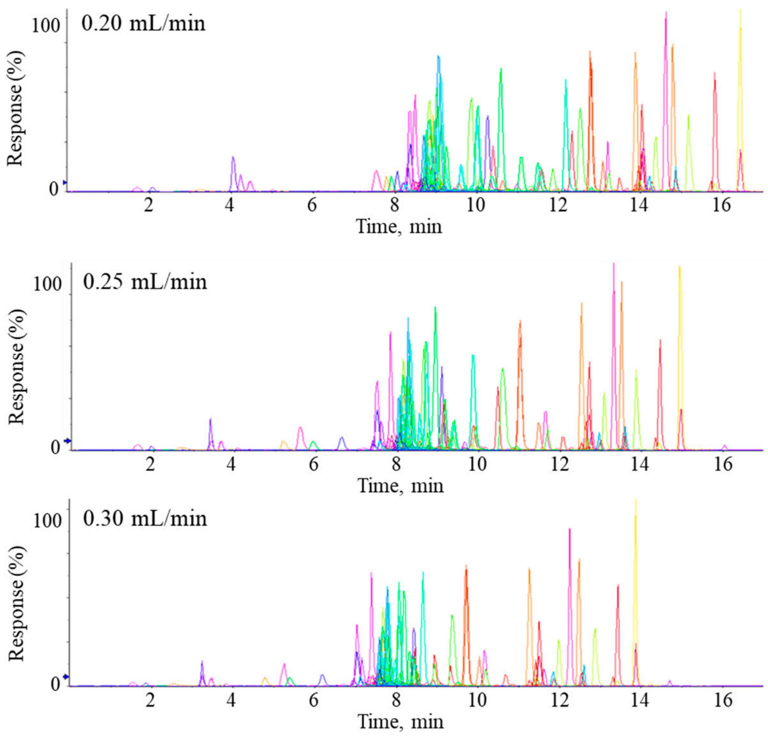

Figure S2. Extracted ion chromatograms in optimization of flow rate

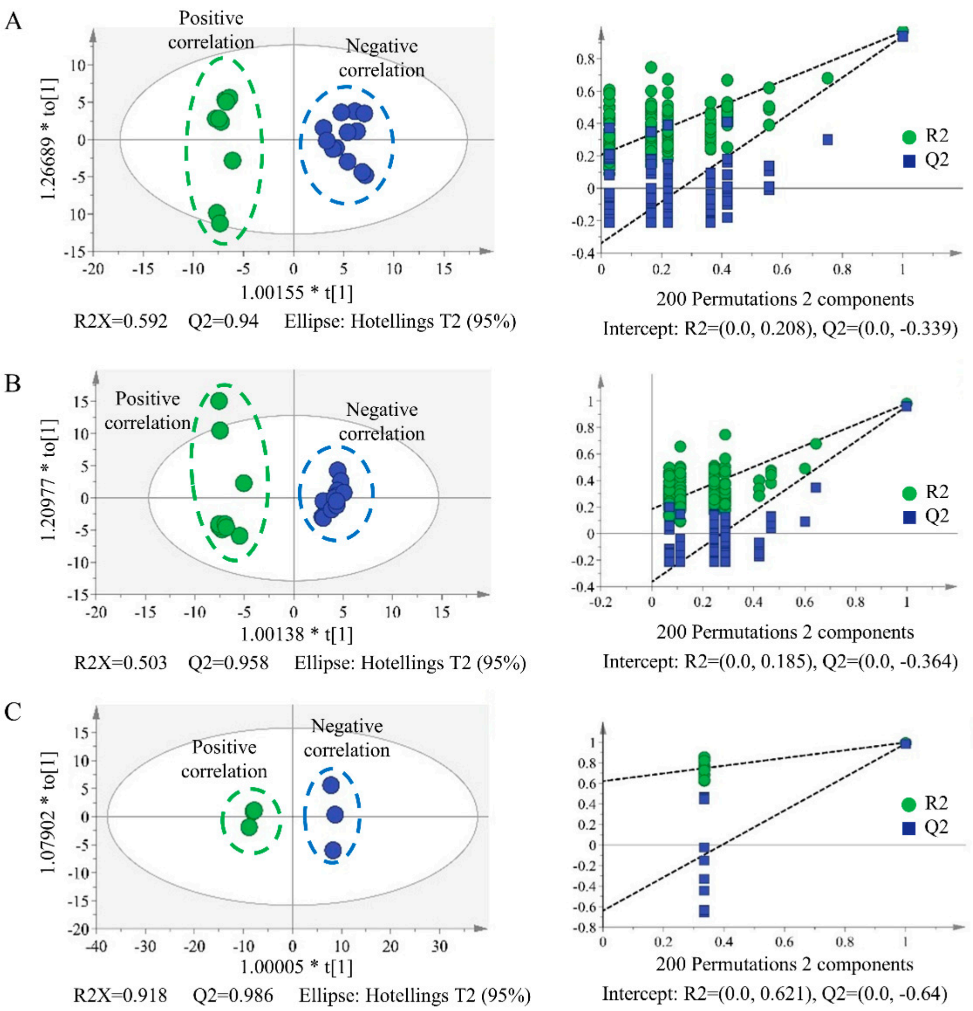

Figure S3. The OPLS-DA models constructed for screening geographically sensitive chemical components and the corresponding results of permutation tests. (A) Latitude; (B) Longitude; (C) Altitude.

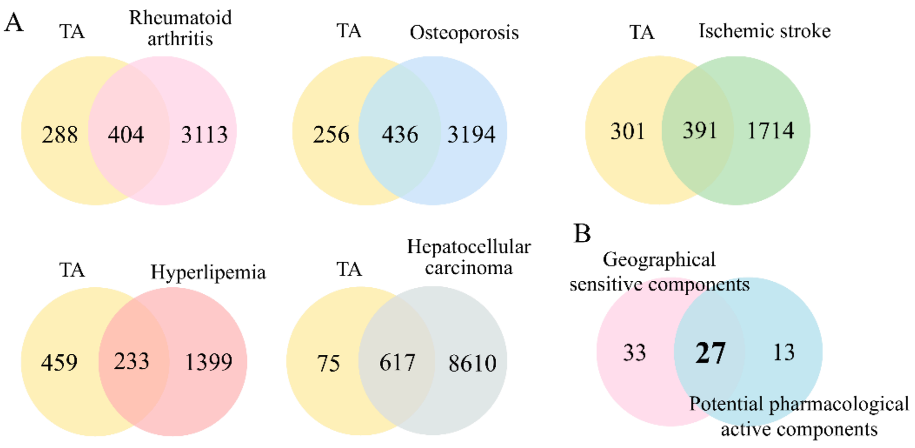

Figure S4. The Venn diagram related to Q-Marker selection. (A) Target intersect of 40 components in Toddaliae Asiaticae Radix (TA) and five diseases; (B) Intersect of 60 geographically sensitive components and 40 potential pharmacological active compounds.

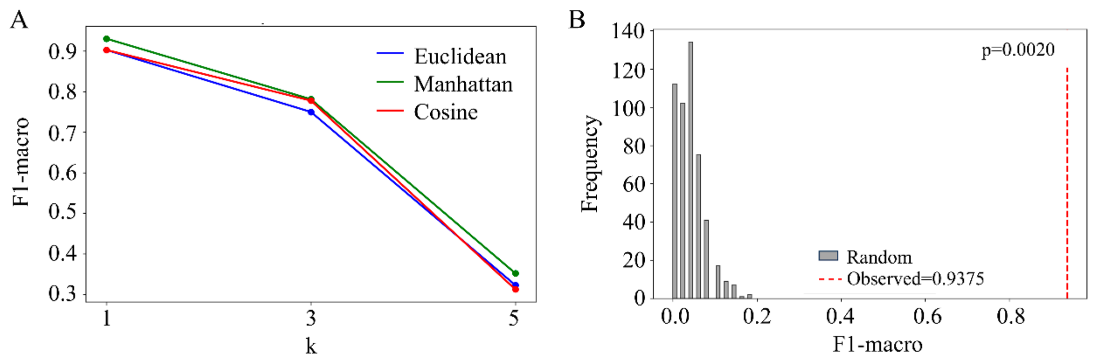

Figure S5. Optimization and assessment of KNN based region classification model using 27 Q-Markers. (A) Optimization of K value; (B) Permutation verification with 500-iteration.

Table S1. Detail information of collection region of medicinal plant *Toddalia asiatica*. (Cfa: humid subtropical climate; Cwa: monsoon-influenced humid subtropical climate; Cwb: subtropical highland climate with dry winter; Am: tropical monsoon climate)

Code	Site (County/District, City, Province)	Latitude	Longitude	Altitude (m)	Climatic zone	Approx. annual precipitation (mm)	Approx. mean annual temperature (° C)
E1	Dehua, Quanzhou, Fujian (D-FJ)	25.50° N	118.15° E	683	Cfa	1416–1873	18.2–18.7
E2	Youxi, Sanming, Fujian (Y-FJ)	26.01° N	117.96° E	571	Cfa	1427–1856	17.9–18.4
E3	Xunwu, Ganzhou, Jiangxi (X-JX)	25.20° N	115.65° E	679.6	Cfa	1255–2097	19.3–19.4
E4	Tonggu, Yichun, Jiangxi (T-JX)	28.58° N	114.29° E	582	Cfa	1535–2011	17.2–17.8
E5	Conghua, Guangzhou,Guangdong (C-GD)	23.70° N	113.86° E	467	Cfa	2340–2724	22.1–22.3
E6	Jianghua, Yongzhou, Hunan (J-HuN)	24.96° N	112.04° E	377.4	Cfa	1618–2220	17.3–17.5
E7	Lianjiang, Zhanjiang, Guangdong (L-GD)	21.81° N	110.05° E	294.3	Cwa	2176–2343	23.0–23.1
E8	Linshui, Hainan (L-HaiN)	18.61° N	110.03° E	97.9	Am	2352–2752	24.6–25.4
E9	Jinxiu, Laibin, Guangxi (J-GX)	23.94° N	110.01° E	912.1	Cwa	1541–1813	20.6–20.7
E10	Sanya, Hainan (S-HaiN)	18.39° N	109.64° E	202.1	Am	2388–2870	24.0–24.7
E11	Enshi, Hubei (E-HB)	30.11°N	109.37°E	727.3	Cfa	1430–1652	14.7–15.0
E12	Yizhou, Hechi, Guangxi (Y-GX)	24.36° N	108.89° E	269.2	Cwa	1536–1839	20.6–20.6
E13	Xiushan, Chongqing (X-CQ)	28.31°N	108.81°E	631.5	Cwa	1512–1651	16.0–16.3
E14	Xuyong, Luzhou, Sichuan (X-SC)	27.84° N	105.47° E	1191	Cwb	1139–1360	16.2–16.8
E15	Yanjin, Zhaotong, Yunnan (Y-YN)	28.05° N	104.23° E	544	Cwa	1071–1314	15.6–16.5
E16	Hekou, Honghe, Yunnan (H-YN)	22.85° N	104.17° E	1447.5	Cwb	1833–1875	17.1–18.1

Table S2. Detail information of 80 target components in *Toddalia asiatica* for rapid quantitative analytical method.

No.	Components	Formula	Molecular ion	MS（*m/z*）	MS/MS (*m/z*)	Category
1	Adenine	$C_5H_5N_5$	$[M+H]^+$	136.0617	119.0351，109.0508，94.0399，92.0243，67.0291	Others
2	Guanine	$C_5H_5N_5O$	$[M+H]^+$	152.0569	135.0303，128.0456，110.0350，82.0401	Others
3	L-Phenylalanine	$C_9H_{11}NO_2$	$[M+H]^+$	166.0862	149.0597，137.0597，131.0491，123.044，121.0648，112.0393	Others
4	Thymine	$C_5H_6N_2O_2$	$[M+H]^+$	127.0504	110.0238，84.0445，82.0288，81.0448，56.0496，54.0340	Others
5	Indoline	C_8H_9N	$[M+H]^+$	120.0808	103.0542，95.0492，93.0699，77.0386	Others
6	Heliamine	$C_{11}H_{15}NO_2$	$[M+H]^+$	194.1177	177.0911, 145.0648, 117.0699	Alkaloid
7	1,2,3,4-Tetrahydroisoquinoline-3-carboxylic acid	$C_{10}H_{11}NO_2$	$[M+H]^+$	178.0865	165.0719，163.0630，135.0681，69.0336	Others
8	3-Methoxycatechol	$C_7H_8O_3$	$[M+H]^+$	141.0546	126.0313，109.0285，98.0363，95.0491，81.0335，53.0387	Others
9	Neochlorogenic acid	$C_{16}H_{18}O_9$	$[M-H]^-$	353.0875	191.0562，179.0351，173.0454，135.0452	Organic acid
10	Higenamine	$C_{16}H_{17}NO_3$	$[M+H]^+$	272.1279	255.1013，237.0898，161.0595，143.0483，107.0490	Alkaloid
11	3-Methoxyphenylacetic acid	$C_9H_{10}O_3$	$[M+H]^+$	167.0704	123.0441，106.0413，78.0465	Others
12	N-Methyl-14-O-demethylepiporphyroxine	$C_{20}H_{21}NO_6$	$[M+H]^+$	372.1444	327.0864，295.0601，239.0703	Alkaloid

No.	Components	Formula	Molecular ion	MS（*m/z*）	MS/MS (*m/z*)	Category
13	Chlorogenic acid	$C_{16}H_{18}O_9$	$[M-H]^-$	353.0876	191.0559，179.0347，173.0452，135.0448	Organic acid
14	Vanillic acid	$C_8H_8O_4$	$[M-H]^-$	169.0494	151.0388, 136.0177, 123.0423, 93.0335	Organic acid
15	Cryptochlorogenic acid	$C_{16}H_{18}O_9$	$[M-H]^-$	353.0877	191.0557，179.0345，173.0450，135.0446	Organic acid
16	2,4,10-Trimethoxy-5,8-dihydro-6H-isoquinolino[3,2-a]isoquinoline-3,11-diolglucoside	$C_{20}H_{31}NO_{10}$	$[M+H]^+$	518.2016	356.1477, 338.1372, 206.0803, 188.0701, 149.0594	Alkaloid
17	Cyclanoline	$C_{20}H_{24}NO_4^+$	$[M]^+$	342.1702	192.1020，177.0779，151.0748	Alkaloid
18	Phellodendrine	$C_{20}H_{24}NO_4^+$	$[M]^+$	342.1704	192.1015, 177.0775, 149.0834, 120.0764	Alkaloid
19	Coclaurine	$C_{17}H_{19}NO_3$	$[M+H]^+$	286.1443	269.1170, 237.0908, 209.0960, 175.0754,143.0487, 107.0494	Alkaloid
20	Stepharine	$C_{18}H_{19}NO_3$	$[M+H]^+$	298.1439	281.1167, 269.1162, 254.0925, 238.0979, 192.1017, 161.0830	Alkaloid
21	Tembetarine	$C_{20}H_{26}NO_4^+$	$[M]^+$	344.1859	175.0754，143.0492，137.0595，58.0662	Alkaloid
22	Coreximine	$C_{19}H_{21}NO_4$	$[M+H]^+$	328.1545	313.1311，298.1076，166.0865，151.0756	Alkaloid
23	Magnoflorine	$C_{20}H_{24}NO_4^+$	$[M]^+$	342.1703	297.1108, 282.0896, 265.0842, 237.0896, 222.0661, 209.0939, 191.0840, 58.0661	Alkaloid
24	Tryptophol	$C_{10}H_{11}NO$	$[M+H]^+$	162.0916	144.0810，134.0966，120.0809，80.0496	Others

No.	Components	Formula	Molecular ion	MS（*m/z*）	MS/MS (*m/z*)	Category
25	3-O-Feruloylquinic acid	$C_{17}H_{20}O_9$	$[M-H]^-$	367.1033	191.0560，173.0454，134.0373	Organic acid
26	Dehydromagnolialine	$C_{20}H_{23}NO_4^+$	$[M]^+$	340.1544	295.0963，263.0701，236.0753	Others
27	(1,3)Benzodioxolo(5,6-c)phenanthridin-13-ol	$C_{21}H_{19}NO_5$	$[M]^+$	366.1339	321.0996，320.0917，306.0761，291.0891，278.0812，262.0862，200.0706，186.0549	Alkaloid
28	Laurifoline	$C_{20}H_{24}NO_4^+$	$[M]^+$	342.1704	297.1110, 282.0866, 265.0847, 250.0610, 237.0899, 205.0637, 58.0660	Alkaloid
29	Magnocurarine/Isomer	$C_{19}H_{24}NO_3^+$	$[M]^+$	314.175	269.1173，254.0930，237.0910，209.0962，175.0754，143.0493，107.0493，58.0661	Alkaloid
30	N-Methylpalaudinium	$C_{21}H_{27}NO_4$	$[M+H]^+$	358.2012	313.1435，189.0911，137.0598，58.0661	Alkaloid
31	Pterophorine	$C_{20}H_{23}NO_7$	$[M+H]^+$	390.1546	345.0963，327.0858，295.0596，285.0752，147.0073	Alkaloid
32	Umbelliferone	$C_9H_6O_3$	$[M+H]^+$	163.0392	135.0441，119.0493，107.0481，58.0661	Coumarin
33	Dihydrotanshinone I	$C_{18}H_{14}O_3$	$[M+H]^+$	279.1018	264.0783，248.0834，236.0834，219.0806，208.0885，191.0857	Others
34	N-Methyl isocorydine/N-Methyl corydine	$C_{21}H_{26}NO_4^+$	$[M]^+$	356.1863	311.1284，296.1049，279.1019，264.0786，248.0836，236.0838，191.0858，58.0661	Alkaloid
35	Psi-Ribalinine	$C_{15}H_{17}NO_3$	$[M+H]^+$	260.1286	242.1169, 200.0705, 188.0702, 176.0697, 172.0750, 144.0809, 134.0593	Alkaloid
36	Menadiol	$C_{11}H_{10}O_2$	$[M+H]^+$	175.0755	160.0521，147.0806，132.0571，119.0856，117.0700，115.0544，107.0493，91.0543	Others
37	Isofraxidin	$C_{11}H_{10}O_5$	$[M+H]^+$	223.0602	208.0368，190.0262，179.0341，162.0313，135.0442，107.0493	Coumarin

No.	Components	Formula	Molecular ion	MS（*m/z*）	MS/MS (*m/z*)	Category
38	4’-Methoxymagnocurarine	$C_{20}H_{26}NO_3^+$	$[M]^+$	328.1909	283.1331，251.1069，248.0816，236.0833，219.0790，191.0845，58.0661	Alkaloid
39	Formononetin	$C_{16}H_{12}O_4$	$[M+H]^+$	269.0807	254.0572，241.0858，226.0623，209.0596，199.0752，181.0647	Flavonoids
40	Nodakenin	$C_{20}H_{24}O_9$	$[M+H]^+$	409.1491	247.0968，229.0840，187.0385，175.0360	Coumarin
41	Protopine	$C_{20}H_{19}O_5N$	$[M+H]^+$	354.1338	336.1233，275.0706，206.0813，188.0708，175.0392，149.0599	Alkaloid
42	Corynoline	$C_{21}H_{21}NO_5$	$[M]^+$	368.1494	353.1260，352.1579，338.1026，336.1234，324.1230，307.1204	Alkaloid
43	3,4-Dicaffeoylquinic acid	$C_{25}H_{24}O_{12}$	$[M-H]^-$	515.1182	353.0894，353.0872, 191.0569, 179.0360, 135.0457	Organic acid
44	Ribalinine	$C_{15}H_{17}NO_3$	$[M+H]^+$	260.1286	242.1169, 200.0705, 188.0702, 176.0697, 172.0750, 144.0809, 134.0593	Alkaloid
45	Marsupsin	$C_{16}H_{14}O_6$	$[M+H]^+$	303.0866	179.0341，177.0548，153.0184	Flavonoids
46	Allocryptopine	$C_{21}H_{23}NO_5$	$[M+H]^+$	370.1652	352.1546，290.1940，189.0780.188.0708	Alkaloid
47	Hesperidin	$C_{28}H_{34}O_{15}$	$[M-H]^-$	609.1829	301.0715，286.0494，257.0821, 151.003	Flavonoids
48	6,7-Dimethoxychromen-2-one	$C_{11}H_{10}O_4$	$[M+H]^+$	207.0653	191.0357, 179.0674, 151.0742, 135.0491, 107.0486, 91.0563	Coumarin
49	3-Aminopyrrolidine-3-carboxylic acid	$C_5H_{10}N_2O_2$	$[M+H]^+$	131.0815	114.0549，103.0543，90.0551，87.0761	Others
50	Columbamine isomer	$C_{20}H_{20}NO_4^+$	$[M]^+$	338.1393	323.1160，322.1078，308.0922，294.1129	Alkaloid

No.	Components	Formula	Molecular ion	MS（*m/z*）	MS/MS (*m/z*)	Category
51	Berberrubine	$C_{19}H_{16}NO_4^+$	$[M]^+$	322.1078	307.0832，292.0583，279.0889	Alkaloid
52	Columbamine	$C_{20}H_{20}NO_4^+$	$[M]^+$	338.1386	323.1140, 322.1059, 308.0925, 294.1113	Alkaloid
53	Nornitidine	$C_{20}H_{15}NO_4$	$[M+H]^+$	334.1075	319.0840，304.0591，291.0882，276.0644	Alkaloid
54	Haplopine	$C_{13}H_{11}NO_4$	$[M+H]^+$	246.0763	231.0528，216.0284，188.0332, 160.0389	Alkaloid
55	Norchelerythrine	$C_{20}H_{15}NO_4$	$[M+H]^+$	334.1075	319.0822, 304.0591, 291.0880, 276.0644	Alkaloid
56	Marmesin	$C_{14}H_{14}O_4$	$[M+H]^+$	247.0968	229.0862，213.0540，175.0392，147.0438	Coumarin
57	5,6,7-Trimethoxycoumarin	$C_{12}H_{12}O_5$	$[M+H]^+$	237.0759	222.0524，207.0289，193.0498，176.0469，166.0627，151.0756，148.0521	Coumarin
58	Sanguinarine	$C_{18}H_{19}NO_4$	$[M+H]^+$	332.0923	317.0686，304.0970，302.0813，274.0868	Alkaloid
59	Mexoticin/ Toddalolactone	$C_{16}H_{20}O_6$	$[M+H]^+$	309.1335	291.1230, 235.0602, 219.0654, 205.0496, 177.0539, 161.0600	Coumarin
60	2-Phenylphenol	$C_{12}H_{10}O$	$[M+H]^+$	171.0807	153.0701，143.0857，128.0622，115.0544，105.0337，91.0544，79.0544	Others
61	Berberine	$C_{20}H_{18}NO_4^+$	$[M]^+$	336.1238	321.0964, 320.0918, 306.0762, 292.0969, 278.0793	Alkaloid
62	4-Methoxy-1-methyl-2-quinoLone	$C_{11}H_{11}NO_2$	$[M+H]^+$	190.0866	175.0653, 158.0626, 147.0691, 118.0660	Alkaloid
63	Nitidine	$C_{21}H_{18}NO_4^+$	$[M]^+$	348.1233	333.0983, 332.0899, 318.0748, 304.0954, 290.0802	Alkaloid

No.	Components	Formula	Molecular ion	MS（m/z）	MS/MS (m/z)	Category
64	Robustine	$C_{12}H_9NO_3$	$[M+H]^+$	216.0648	201.0426, 183.0311, 173.0456, 155.0341, 145.0493, 127.0405, 117.0550	Alkaloid
65	Chelerythrine	$C_{21}H_{18}NO_4^+$	$[M]^+$	348.1232	333.0978, 332.0893, 318.0738, 304.0950, 290.0798	Alkaloid
66	Skimmianine	$C_{14}H_{13}NO_4$	$[M+H]^+$	260.0919	245.0669, 227.0564, 216.0642, 199.0618, 184.0384, 156.0435, 128.0486, 101.0382	Alkaloid
67	γ-Fagarine	$C_{13}H_{11}NO_3$	$[M+H]^+$	230.0815	215.0569, 200.0335, 186.0543, 172.0389, 158.0593, 144.0438, 116.0489, 89.0391	Alkaloid
68	Hesperetin	$C_{16}H_{14}O_6$	$[M-H]^-$	301.0719	286.0472, 242.0590, 151.0033, 123.0093, 107.0137	Flavonoids
69	Diosmetin	$C_{16}H_{12}O_6$	$[M-H]^-$	299.0561	284.0332, 256.0359, 227.0335, 211.0401, 183.0427, 151.0040, 133.0295, 107.0143	Flavonoids
70	Dictamnine	$C_{12}H_9NO_2$	$[M+H]^+$	200.0707	185.0472, 129.0574, 102.0465	Alkaloid
71	5,6-Dihydro-11-methoxyyangonin	$C_{16}H_{18}O_5$	$[M+H]^+$	291.1229	259.0968，220.0731，205.0498，191.0705，174.0678	Others
72	Isoarnottianamide	$C_{21}H_{19}NO_6$	$[M+H]^+$	382.1286	364.1163, 349.0946, 339.1054, 292.0704	Alkaloid
73	Luvangetin	$C_{15}H_{14}O_4$	$[M+H]^+$	259.0963	243.0672, 229.0545, 227.0720, 201.0516,173.0591, 161.0630, 141.0694, 128.0613,127.0531, 115.0522	Coumarin
74	5-Hydroxyxanthotoxin	$C_{12}H_8O_5$	$[M+H]^+$	233.0446	218.0210，190.0262，162.0313，134.0363	Coumarin
75	Arnottianamide	$C_{21}H_{19}NO_6$	$[M+H]^+$	382.1288	364.1261, 354.1376, 339.1126, 292.0719	Alkaloid

No.	Components	Formula	Molecular ion	MS（*m/z*）	MS/MS (*m/z*)	Category
76	N-Methylflindersine	$C_{15}H_{15}NO_2$	$[M+H]^+$	242.1175	200.0704, 188.0704, 172.0764, 144.0805	Alkaloid
77	Oxychel1erythrine	$C_{21}H_{17}NO_5$	$[M+H]^+$	364.1186	349.0938, 348.0863, 334.0696, 320.0896, 306.0756, 301.0743, 278.0763	Alkaloid
78	7-Methyl ether heteropeucenin	$C_{16}H_{18}O_4$	$[M+H]^+$	275.1276	245.0809, 217.0500, 205.0489, 189.0521	Flavonoids
79	Norchelerythrine Isomer	$C_{20}H_{15}NO_4$	$[M+H]^+$	334.1076	319.0821, 318.0744, 304.0605, 302.0800, 291.0882, 290.0797, 276.0654	Alkaloid
80	Dihydrochelerythrine	$C_{21}H_{19}NO_4$	$[M+H]^+$	350.1385	335.1137, 334.1059, 318.0984, 304.0945	Alkaloid

Table S3. The optimized parameters of UHPLC-MRM MS/MS method for targeted analysis of components in *T. asiatica*

No.	Components	Q1 (*m/z*)	Q3 (*m/z*)	DP (V)	CE (eV)	Retention time (min)
1	Indoline	120.1	103.1	50	25	7.25
2	Thymine	127.1	110.0	50	20	7.26
3	3-Aminopyrrolidine-3-carboxylic acid	131.1	103.1	70	20	8.29
4	Adenine	136.1	119.0	50	30	2.75
5	3-Methoxycatechol	141.1	81.0	50	30	7.13
6	Guanine	152.1	135.0	90	25	6.01
7	Tryptophol	162.1	120.1	90	25	8.65
8	Umbelliferone	163.0	135.0	50	20	10.56
9	L-Phenylalanine	166.1	149.1	50	20	3.51
10	3-Methoxyphenylacetic acid	167.1	123.0	50	30	7.38
11	Vanillic acid	169.0	151.0	50	20	8.92
12	2-Phenylphenol	171.1	143.1	90	20	8.93
13	Menadiol	175.1	147.1	70	20	7.78
14	1,2,3,4-Tetrahydroisoquinoline-3-carboxylic acid	178.1	163.1	70	30	6.64
15	4-Methoxy-1-methyl-2-quinoLone	190.1	175.1	70	30	5.08

No.	Components	Q1 (*m/z*)	Q3 (*m/z*)	DP (V)	CE (eV)	Retention time (min)
16	Heliamine	194.1	177.1	70	20	6.26
17	Dictamnine	200.1	185.0	50	35	11.51
18	6,7-Dimethoxychromen-2-one	207.1	179.1	70	35	11.29
19	Robustine	216.1	201.0	70	30	8.27
20	Isofraxidin	223.1	208.0	50	40	8.96
21	γ -Fagarine	230.1	215.1	50	45	10.45
22	5-Hydroxyxanthotoxin	233.0	205.0	70	25	8.65
23	5,6,7-Trimethoxycoumarin	237.1	222.1	50	25	10.38
24	N-Methylflindersine	242.1	200.1	70	30	14.09
25	Haplopine	246.1	231.1	90	20	9.20
26	Marmesin	247.1	229.1	110	20	9.40
27	Luvangetin	259.1	236.1	110	25	11.98
28	Skimmianine	260.1	245.1	50	35	10.26
29	Ribalinine	260.1	200.1	50	30	2.02
30	Psi-Ribalinine	260.1	242.1	50	30	2.34
31	Formononetin	269.1	254.1	90	30	7.69
32	Higenamine	272.1	255.1	70	20	7.45

No.	Components	Q1 (*m/z*)	Q3 (*m/z*)	DP (V)	CE (eV)	Retention time (min)
33	7-Methyl ether heteropeucenin	275.1	219.1	50	25	15.20
34	Dihydrotanshinone I	279.1	264.1	90	25	7.71
35	Coclaurine	286.1	269.1	50	20	7.61
36	5,6-Dihydro-11-methoxyyangonin	291.1	220.1	50	45	13.15
37	Stepharine	298.1	283.1	110	35	7.58
38	Diosmetin	299.1	284.0	50	30	7.59
39	Hesperetin	301.1	286.0	110	35	8.45
40	marsupsin	303.1	177.1	50	40	7.93
41	Mexoticin/ Toddalolactone	309.1	291.1	70	25	8.94
42	Magnocurarine/Isomer	314.2	107.0	50	50	7.68
43	Berberrubine	322.1	307.1	50	35	7.92
44	Coreximine	328.2	298.1	90	40	7.57
45	4’-Methoxymagnocurarine	328.2	121.1	70	30	7.75
46	Sanguinarine	332.1	274.1	70	45	8.07
47	Nornitidine	334.1	319.1	70	40	7.99
48	Norchelerythrine	334.1	318.1	70	40	8.01

No.	Components	Q1 (*m/z*)	Q3 (*m/z*)	DP (V)	CE (eV)	Retention time (min)
49	Norchelerythrine Isomer	334.1	291.1	70	40	8.01
50	Berberine	336.1	320.1	70	40	8.15
51	Columbamine	338.1	322.1	70	30	7.93
52	Columbamine isomer	338.1	308.1	70	30	7.93
53	Dehydromagnolialine	340.2	295.1	70	35	7.63
54	Magnoflorine	342.2	297.1	50	25	7.59
55	Cyclanoline	342.2	192.1	50	35	7.56
56	Phellodendrine	342.2	177.1	50	25	7.60
57	Laurifoline	342.2	205.1	50	25	7.57
58	Tembetarine	344.2	299.1	70	30	7.62
59	Chelerythrine	348.1	333.1	50	25	8.15
60	Nitidine	348.1	318.1	50	25	8.15
61	Dihydrochelerythrine	350.1	147.0	50	35	7.46
62	Neochlorogenic acid	353.1	173.0	70	55	7.86
63	Chlorogenic acid	353.1	179.0	70	55	8.04
64	Cryptochlorogenic acid	353.1	191.1	70	55	7.90
65	Protopine	354.1	336.1	70	25	7.83

No.	Components	Q1 (*m/z*)	Q3 (*m/z*)	DP (V)	CE (eV)	Retention time (min)
66	N-Methyl isocorydine/N-Methyl corydine	356.2	311.1	70	35	7.71
67	N-Methylpalaudinium	358.2	313.1	50	35	7.64
68	Oxychel1erythrine	364.1	349.1	50	25	14.01
69	[1,3]Benzodioxolo[5,6-c]phenanthridin-13-ol	366.1	350.1	70	50	7.74
70	3-O-Feruloylquinic acid	367.1	191.1	70	25	5.02
71	Corynoline	368.1	353.1	70	35	7.83
72	Allocryptopine	370.2	188.1	70	35	7.91
73	N-Methyl-14-O-demethylepiporphyroxine	372.1	327.1	50	20	7.59
74	Arnottianamide	382.1	354.1	70	25	12.23
75	Isoarnottianamide	382.1	349.1	70	25	10.63
76	Pterophorine	390.2	345.1	70	30	7.68
77	Nodakenin	409.1	247.1	70	25	8.94
78	3,4-Dicaffeoylquinic acid	515.1	353.1	50	25	8.69
79	2,4,10-Trimethoxy-5,8-dihydro-6H-isoquinolino[3,2-a]isoquinoline-3,11-diolglucoside	518.2	356.1	50	60	7.49

No.	Components	Q1 (*m/z*)	Q3 (*m/z*)	DP (V)	CE (eV)	Retention time (min)
80	Hesperidin	609.2	301.1	50	25	8.61

Table S4. Methodological validation results of quantitative analysis for components in *T. asiatica*

Q1 (*m/z*)	Q3 (*m/z*)	Linear equation	r^2	Precision（RSD, %）		Repeatability（RE, %）			Stability（%）
				Inter-day	Intra-day	L	M	H	
120.1	103.1	y=1.38e^8x+42900	0.9903	5.67	3.81	6.82	5.68	7.87	3.81
127.1	110.0	y=5.05e^6x+10289.97	0.9946	9.09	10.98	7.98	5.24	8.98	10.98
131.1	103.1	y=1.35e^8x+7180.86	0.9910	14.38	12.32	10.34	11.29	13.67	12.32
136.1	119.0	y=1.48e^8x+3709.03	0.9918	6.34	7.46	13.98	13.64	12.98	7.46
141.1	81.0	y=1.77e^6x+26297.42	0.9902	9.08	13.98	8.67	9.86	7.54	13.98
152.1	135.0	y=3.09e^8x+26297.42	0.9902	15.67	14.86	7.98	10.23	9.58	14.86
162.1	120.1	y=1.03e^6x+11504.22	0.9916	16.34	15.78	11.25	10.84	12.57	15.78
163.0	135.0	y=1.82e^8x+1.54e^5	0.9906	9.18	9.63	2.59	12.33	3.43	9.63
166.1	149.1	y=6.84e^7x+15406.76	0.9908	9.86	10.34	3.46	8.58	4.67	7.55
167.1	123.0	y=7.37e^7x+1239.80	0.9914	6.98	7.38	4.67	10.56	5.34	8.35
169.0	151.0	y=1.45e^8x+13377.51	0.9948	10.14	11.63	7.25	5.35	12.16	11.63
171.1	143.1	y=7.98e^5x+24511.77	0.9905	3.45	5.56	2.33	4.54	3.24	5.56
175.1	147.1	y=7.71e^6x+15377.43	0.9919	16.21	15.96	12.98	14.87	13.22	15.96
178.1	163.1	y=5.20e^7x+48000	0.9932	4.38	5.87	6.93	5.68	7.42	5.87
190.1	175.1	y=6.87e^8x+68300	0.9904	5.33	5.54	3.31	5.5	3.22	5.54
194.1	177.1	y=9.72e^7x+1.04e^6	0.9917	0.58	2.24	7.1	0.68	7.52	2.24
200.1	185.0	y=5.53e^8x+-30230.7	0.9925	1.24	7.78	7.21	1.14	1.11	7.78

Q1 (*m/z*)	Q3 (*m/z*)	Linear equation	r^2	Precision（RSD, %）		Repeatability（RE, %）			Stability（%）
				Inter-day	Intra-day	L	M	H	
207.1	179.1	y=3.52e^8x+101358	0.9948	8.54	12.43	0.15	7.7	13.31	12.43
216.1	201.0	y=4.46e^7x+143802	0.9913	5.52	9.62	4.09	5.61	14.83	9.62
223.1	208.0	y=1.87e^8x+50383.6	0.9903	0.18	5.67	9.57	0.41	14.56	5.67
230.1	215.1	y=1.97e^8x+1863240	0.9938	1	0.15	1.24	4.24	11.87	0.15
233.0	205.0	y=9.83e^7x+6178.58	0.9959	0.74	0.95	8.9	9.53	7.07	0.95
237.1	222.1	y=7.65e^9x+1250960	0.9907	3.1	2.22	11.5	4.16	4.17	2.22
242.1	200.1	y=1.65e^7x+38362	0.9904	5.18	1.71	11.37	6.16	1.56	1.71
246.1	231.1	y=1.21e^8x+7230.25	0.9932	0.95	6.48	2.35	7.21	14.53	6.48
247.1	229.1	y=2.82e^8x+5463.41	0.9910	4.91	0.55	5.99	4.96	13.01	0.55
259.1	236.1	y=4.65e^8x+-17410.25	0.9967	1.83	1.84	2.58	3.64	1.96	1.84
260.1	245.1	y=1.45e^8x+10741.25	0.9920	2.71	1.52	1.22	4.22	11.18	1.52
260.1	178.2	y=2.80e^6x+-535.71	0.9971	4.27	12.07	8.27	4.46	14.47	12.07
260.1	242.1	y=1.08e^8x+-825952	0.9972	8.89	12.99	14.48	4.11	12.65	12.99
269.1	254.1	y=2.66e^7x+13388.86	0.9955	6.96	9.14	0.42	2.21	13.69	9.14
272.1	255.1	y=4.79e^7x+5105.36	0.9925	2.73	5.74	1.80	2.87	2.35	5.74
275.1	219.1	y=1.67e^7x+-2845.68	0.9991	0.33	0.02	0.25	3.22	4.06	0.02
279.1	264.1	y=2.56e^6x+15141.56	0.9911	4.34	5.41	2.47	3.26	3.84	5.41
286.1	269.1	y=1.64e^8x+94985	0.9915	4.12	13.24	14.34	8.43	3.95	13.24

Q1 (*m/z*)	Q3 (*m/z*)	Linear equation	r^2	Precision（RSD, %）		Repeatability（RE, %）			Stability（%）
				Inter-day	Intra-day	L	M	H	
291.1	220.1	y=7.69e^7x+14121.72	0.9935	5.95	3.48	1.81	6.34	10.67	3.48
298.1	283.1	y=4.295e^8x+38356.7	0.9915	5.88	8.18	3.57	4.70	11.99	8.18
299.1	284.0	y=1.35e^8x+7180.86	0.9910	8.30	8.36	2.68	4.34	10.76	8.36
301.1	286.0	y=7.31e^7x+582425	0.9923	14.83	14.32	3.76	4.7	2.66	14.32
303.1	153.0	y=1.68e^8x+5838.57	0.9924	12.26	13.55	2.16	11.47	14.59	13.55
303.1	177.1	y=1.25e^8x+7742.97	0.9923	5.24	6.59	3.00	9.20	14.61	6.59
309.1	291.1	y=1.13e^9x+74665.9	0.9924	7.40	14.72	1.38	6.13	14.83	14.72
314.2	107.0	y=1.95e^9x+4.03	0.9928	12.79	14.36	0.14	2.66	12.98	14.36
322.1	307.1	y=1.58e^7x+4263.15	0.9920	3.21	4.09	5.49	8.75	5.51	4.09
328.2	298.1	y=1.19e^9x+23198.38	0.9941	5.29	5.07	5.94	0.75	5.94	5.07
328.2	121.1	y=1.02e^9x+-68301.8	0.9932	1.20	6.89	12.21	8.67	14.44	6.89
332.1	274.1	y=4.07e^9x+-48943.5	0.9910	7.87	3.98	5.61	1.72	4.11	3.98
332.1	314.1	y=1.15e^7x+1249.36	0.9914	1.39	4.94	2.68	5.69	13.53	4.94
334.1	319.1	y=2.91e^9x+61618.7	0.9968	6.27	5.29	12.47	3.40	7.21	5.29
334.1	318.1	y=2.6e^8x+-14814.66	0.9902	10.92	13.41	13.76	13.69	3.44	13.41
336.1	320.1	y=1.19e^8x+-99896.1	0.9963	3.27	1.24	13.39	8.21	11.06	1.24
338.1	322.1	y=1.97e^8x+-3814.09	0.9920	1.00	1.22	2.35	6.93	6.94	1.22
340.2	295.1	y=1.43e^8x+-1512.94	0.9981	3.11	11.8	3.10	7.41	13.55	11.80

Q1 (*m/z*)	Q3 (*m/z*)	Linear equation	r^2	Precision（RSD, %）		Repeatability（RE, %）			Stability（%）
				Inter-day	Intra-day	L	M	H	
342.2	297.1	y=2.64e^9x+1159240	0.9953	0.48	4.90	3.47	6.27	12.42	4.90
342.2	192.1	y=2.47e^9x+-106067	0.9929	3.32	9.69	1.86	1.20	12.15	9.69
342.2	177.1	y=2.68e^8x+5838.57	0.9924	12.26	13.55	2.16	11.47	14.59	13.55
342.2	205.1	y=2.31e^8x+2397.82	0.9932	0.27	10.56	6.52	2.01	14.05	10.56
344.2	299.1	y=4.21e^9x+18867.18	0.9916	3.43	8.41	3.58	1.35	13.3	8.41
348.1	333.1	y=4.89e^8x+-16626.27	0.9951	7.22	1.56	13.82	13.60	11.79	1.56
348.1	318.1	y=4.89e^8x+-16626.27	0.9951	7.22	1.56	13.82	13.60	11.79	1.56
350.1	147.0	y=3.73e^5x+-713.97	0.9905	3.61	0.97	3.33	2.94	4.82	0.97
353.1	173.0	y=1.64e^9x+-15243.36	0.9950	7.56	3.73	4.05	2.23	3.45	3.96
353.1	179.0	y=1.45e^9x+-16297.94	0.9948	7.33	3.85	4.78	1.91	4.17	3.85
353.1	191.1	y=1.56e^9x+-14758.23	0.9938	7.45	3.96	4.68	2.45	4.76	3.57
354.1	336.1	y=3.86285e^8x+6807.43	0.9907	5.20	10.95	5.34	0.96	1.60	10.95
356.2	311.1	y=3.07e^8x+-10152.19	0.9978	7.05	10.79	1.14	3.72	13.49	10.79
358.2	313.1	y=5.97e^8x+27999.10	0.9936	6.94	14.25	3.60	2.14	13.52	14.25
364.1	349.1	y=2.22e^7x+844.98	0.9925	8.96	9.04	4.26	1.75	9.6	9.04
366.1	350.1	y=8.48e^6x+-962.6	0.9956	7.97	13.77	4.97	2.22	1.11	13.77
367.1	191.1	y=5.02e^7x+9409.27	0.9954	9.48	11.20	12.93	0.20	4.59	11.20
368.1	353.1	y=6.68e^8x+-40777	0.9904	4.18	10.90	6.38	6.26	1.75	10.90

Q1 (*m/z*)	Q3 (*m/z*)	Linear equation	r^2	Precision（RSD, %）		Repeatability（RE, %）			Stability（%）
				Inter-day	Intra-day	L	M	H	
370.2	188.1	y=5.75e^8x+-45710	0.9907	6.24	6.41	4.34	8.15	3.03	6.41
372.1	327.1	y=3.56e^7x+17843.70	0.9913	7.35	12.15	8.54	12.13	1.06	12.15
382.1	354.1	y=4.34e^7x+-82806.2	0.9910	3.69	1.38	4.21	4.14	1.29	1.38
382.1	349.1	y=1.56e^8x+2022.37	0.9904	4.17	7.77	11.98	3.80	5.07	7.77
390.2	345.1	y=1.78e^9x+60848.8	0.9947	2.55	6.26	5.32	1.03	14.55	6.26
409.1	247.1	y =2.5e^8x+10646.61	0.9975	11.14	13.51	11.18	6.04	10.82	13.51
515.1	353.1	y=1.32e^7x+67.45	0.9912	1.61	5.55	3.38	6.98	2.02	5.55
518.2	356.1	y=4.42e^6x+4015.74	0.9917	7.53	13.92	0.34	6.05	1.05	13.92
609.2	301.1	y=3.1e^{8}x+26297.42	0.99016	11.20	7.13	5.17	5.50	4.20	7.13

Table S5. 60 chemical components in TA that are correlated with latitude, longitude and altitude. (^{Q} indicates selected Q-Markers)

**Component**	**Latitude**		**Longitude**		**Altitude**	
	**Positive**	**Negative**	**Positive**	**Negative**	**Positive**	**Negative**
Thymine		√				
Menadiol		√				
Stepharine ^{Q}		√				
Cyclanoline ^{Q}		√				
Phellodendrine		√				
Neochlorogenic acid		√				
Chlorogenic acid ^{Q}		√				
Cryptochlorogenic acid		√				
Protopine ^{Q}		√				
N-Methyl isocorydine/N-Methyl corydine		√				
N-Methylpalaudinium		√				
[1,3]Benzodioxolo[5,6-c]phenanthridin-13-ol		√				
Isoarnottianamide		√				
2,4,10-Trimethoxy-5,8-dihydro-6H-isoquinolino[3,2-a]isoquinoline-3,11-diolglucoside		√				
Formononetin ^{Q}			√			
Diosmetin ^{Q}				√		
Nornitidine			√			
Norchelerythrine ^{Q}			√			
Pterophorine ^{Q}			√			
Nodakenin			√			
Indoline						√
3-Aminopyrrolidine-3-carboxylic acid						√
Adenine						√
4-Methoxy-1-methyl-2-quinoLone						√
Heliamine						√
Dictamnine ^{Q}						√
γ-Fagarine ^{Q}						√
5-Hydroxyxanthotoxin ^{Q}						√
Haplopine						√
Marmesin					√	
Higenamine ^{Q}					√	
7-Methyl ether heteropeucenin						√
Coclaurine ^{Q}					√	
5,6-Dihydro-11-methoxyyangonin						√
Berberrubine ^{Q}						√

Component	Latitude		Longitude		Altitude	
	Positive	Negative	Positive	Negative	Positive	Negative
Berberine [Q]						√
Columbamine [Q]						√
Chelerythrine [Q]						√
Dihydrochelerythrine [Q]						√
3-O-Feruloylquinic acid [Q]						√
Corynoline					√	
Tembetarine [Q]		√		√		
Coreximine [Q]		√			√	
Sanguinarine		√			√	
Umbelliferone [Q]		√			√	
L-Phenylalanine		√			√	
Vanillic acid [Q]	√					√
1,2,3,4-Tetrahydroisoquinoline-3-carboxylic acid		√				√
Luvangetin		√				√
Skimmianine [Q]		√				√
Oxychellerythrine		√				√
3-Methoxycatechol			√		√	
6,7-Dimethoxychromen-2-one			√			√
Robustine [Q]			√			√
Isofraxidin [Q]			√		√	
5,6,7-Trimethoxycoumarin			√		√	
Hesperetin [Q]				√		√
Marsupsin				√		√
Mexoticin/Toddalolactone		√		√		√
Magnocurarine/Isomer		√	√		√	

Table S6. Oral bioavailability (OB) and drug-likeness (DL) of 40 potential pharmacologically active components retrieved from the TCMSP database. (compounds satisfying OB ≥ 30% and DL ≥ 0.18 are shown in bold; [Q] indicates selected Q-Markers)

Name	OB	DL
3-O-Feruloylquinic acid [Q]	25.51	0.36
5-Hydroxyxanthotoxin [Q]	NA	NA
6,7-dimethoxycoumarin	NA	NA
Allocryptopine	25.53	0.72
Avicine	NA	NA
**Berberine** [Q]	**36.86**	**0.78**
**Berberrubine** [Q]	**36.74**	**0.73**
**Chelerythrine** [Q]	**43.18**	**0.78**
Chlorogenic acid [Q]	11.93	0.33
Coclaurine [Q]	NA	NA
Columbamine [Q]	26.94	0.59
Coreximine [Q]	NA	NA
Cyclanoline [Q]	2.64	0.57
Dictamnine [Q]	31.39	0.11
**Dihydrochelerythrine** [Q]	**32.73**	**0.81**
**Diosmetin** [Q]	**31.14**	**0.27**
**Formononetin** [Q]	**69.67**	**0.21**
**Hesperetin** [Q]	**70.31**	**0.27**
Hesperidin	13.34	0.67
Heteropeucenin 7-methyl ether	NA	NA
Higenamine [Q]	NA	NA
Isochlorogenic acid B	1.78	0.69
Isocorydine N-oxide	NA	NA
Isofraxidin [Q]	52.32	0.1
Magnocurarine	NA	NA
Magnoflorine	26.69	0.55
Nitidine	18,58	0.81
Nitidine chloride	18.58	0.81
**N-Methylflindersine**	**32.36**	**0.18**
Norchelerythrine [Q]	26.66	0.78
**Oxychelerythrine**	**44.22**	**0.84**
Protopine [Q]	NA	NA
Pterophorine [Q]	NA	NA
Robustine [Q]	NA	NA
**Skimmianine** [Q]	**40.14**	**0.2**
**Stepharine** [Q]	**31.55**	**0.33**
Tembetarine [Q]	2.82	0.36
Umbelliferone [Q]	27.37	0.05
Vanillic acid [Q]	35.47	0.04
γ-Fagarine [Q]	NA	NA
